# Rapid Discrimination of Malignant Breast Lesions from Normal Tissues Utilizing Raman Spectroscopy System: A Systematic Review and Meta-Analysis of In Vitro Studies

**DOI:** 10.1371/journal.pone.0159860

**Published:** 2016-07-26

**Authors:** Ke Deng, Chenjing Zhu, Xuelei Ma, Hongyuan Jia, Zhigong Wei, Yue Xiao, Jing Xu

**Affiliations:** 1 State Key Laboratory of Biotherapy and Cancer Center, West China Hospital, Sichuan University, Chengdu, Sichuan, PR China; 2 West China Hospital, Sichuan University, Chengdu, Sichuan, PR China; School of Medicine, Fu Jen Catholic University, TAIWAN

## Abstract

**Purpose:**

The aim of this study is to evaluate the diagnostic accuracy of Raman spectroscopy system in the detection of malignant breast lesions through a systemic review and meta-analysis of published studies.

**Methods:**

We conducted a comprehensive literature search of PubMed and Embase from 2000 to June 2015. Published studies that evaluated the diagnostic performance of Raman spectroscopy in distinguishing malignant breast lesions from benign lesions and normal tissues were included in our study. The pooled sensitivity, specificity, diagnostic odds ratio, and the area under the curve of summary receiver-operating characteristic curves was derived. A Revised Tool for the Quality Assessment of Diagnostic Accuracy Studies guidelines was used to assess the quality of included studies.

**Results:**

The initial search produced a total of 157 articles after removing duplicates. Nine studies (8 in vitro and 1 in vivo) were eligible in this meta-analysis. We analyzed the eight in vitro studies with 1756 lesions, the pooled sensitivity and specificity of Raman spectroscopy system for the diagnosis of malignant breast lesions were 0.92 (95% CI 0.86–0.96) and 0.97 (97% CI 0.93–0.98), respectively. Diagnostic odds ratio was 266.70 (95% CI 89.38–795.79), and the area under the curve of summary receiver-operating characteristic curves was 0.98 (95% CI 0.97–0.99). Significant heterogeneity was found between studies. There was no evidence of considerable publication bias.

**Conclusions:**

Raman spectroscopy system is an optical diagnostic technology with great value for detecting malignant breast lesions. At the same time, it has advantages of being non-invasive, real-time, and easy to use. Thus it deserves to be further explored for intra-operatory breast tumor margin detection.

## Introduction

Breast cancer is the most prevalent cancer among women worldwide, with an estimated 1.7 million cases and 521,900 deaths in 2012 [1]. The first-line treatment of breast cancer involves breast conserving surgery (BCS) or mastectomy. With the advantages of minimizing volume deficit and maximizing aesthetics, BCS could earn the same long-term survival as mastectomy if tumor-free margin is achieved and followed with radiation to the breast [2]. Margin status is an important prognostic factor for local recurrence. Published rates of positive margins after partial mastectomy vary from 4% to 31% [3], and an increasing number of BCS-eligible women with breast cancer tend to choose mastectomy because many researchers believe mastectomy is more likely to avoid cancer recurrence [4]. According to a consensus guideline on margins in BCS, the use of no ink on tumor as the criterion for an adequate margin in invasive cancer is related to low rates of ipsilateral breast tumor recurrence (IBTR) [5]. However, current radiological and pathological techniques for achieving clear operative margins all have technical and practical limitations [6]. They are often invasive, time-consuming, not accurate enough or prone to subjective interpretations. Therefore, a new detection method with practical advances is needed.

Raman spectroscopy, which measures inelastic scattering of a photon and possesses the ability to probe cellular physiology and tissue physiology at sub-micron length scales conveniently, has been widely used as an analytical tool in many research fields [7, 8]. Recently, Raman spectroscopy system (RAS) has also been explored for biomedical applications (e.g. cancer diagnosis) with its non-invasive and effective features of providing detailed spectroscopic information about biomolecular structures and conformations of cells and tissues [9, 10]. According to the preliminary findings, RAS has shown tremendous promise for mini- or non-invasive, real-time, bedside and intra-operatory cancer discrimination, as well as for an ex vivo imaging tool in support to pathologists. Thus, accurate and thorough classification of breast lesions can be achieved in seconds by applying it to BCS, thereby reducing the number of excisional breast biopsies that are performed and lessening the need for re-excision surgeries resulting from positive margins. However, these previous studies were inconclusive because of mono-centric, inadequate samples and different diagnostic algorithms employed. In this study, we aimed to systematically assess the diagnostic performance of RAS in the rapid differentiation of benign and malignant breast lesions.

## Material and Methods

### Literature search strategy

All studies investigating the diagnostic performance of RAS for breast cancer published from 2000 to June 2015 were identified by searching PubMed and Embase comprehensively. The initial search was performed by combinations of the relevant medical subject heading (MeSH) terms, key words, and word variants for “breast”, “cancer”, “Raman spectra” and “sensitivity” (S1 Appendix). No restriction of language or study type was applied when initial literature searching was conducted. The “related articles” function was used and the reference list was checked to perform the search of all relevant studies, abstracts, and citations.

### Inclusion and exclusion criteria

We screened the full texts of all the related studies to decide whether they were really eligible. Studies included in our meta-analysis had to meet the following criteria: (1) studies focused on the diagnostic value of Raman spectra for breast cancer tissues, (2) studies investigated human breast tissues with histological findings as the gold standard of lesion diagnosis, (3) studies presented sufficient data to construct a 2×2 table corresponding to true positives (TP), true negatives (TN), false positives (FP) and false negatives (FN) directly or indirectly, and (4) studies were published in English.

Exclusion criteria: (1) studies that involved nonhuman subjects, (2) studies without a control group including case reports and case series, (3) reviews or duplicate reports.

### Data extraction

The following data were extracted independently by two reviewers including: the first author’s name, publication year, geographical location, number of patients, number of spectra, sample type (fresh tissue or frozen tissue), methodological and technical data such as diagnostic algorithm, study design (pathologic subtypes of control and case groups), laser wavelength and some other information. TP, TN, FP, and FN were also collected directly or calculated according to the sensitivity, specificity, PPV (Positive Predictive Value) and NPV (Negative Predictive Value) in each reported study.

### Statistical analysis

The diagnostic accuracy of Raman spectra for malignant breast lesions was assessed by calculating pooled sensitivity, specificity, positive likelihood ratio (PLR), and negative likelihood ratios (NLR) values along with corresponding 95% confidence interval (CI). In addition, a summary receiver-operating characteristic (SROC) curve was constructed by the Moses-Shapiro-Littenberg method as a way to investigate the influence of thresholds on consequences [11]. Thresholds might not have an impact on results as the SROC curves were not shoulder-like. For further exploring the potential sources of heterogeneity, the inconsistency index (I^2^) statistic and Chi-square test were applied to perform subgroup analysis. I^2^ > 50% and P value < 0.05 were considered significant for heterogeneity [12]. If heterogeneity existed, a fixed effects model was used to pool parameters among studies [13]. Otherwise, a random effects model was applied. We also conducted Deeks ' funnel plot asymmetry test to investigate publication bias [14]. All the above statistical analysis were performed using STATA 12.0 and Meta-Disc Version 1.4 [15].

### Quality Assessment

To systematically assess the quality of the studies included in this meta-analysis, a revised tool for the Quality Assessment of Diagnostic Accuracy Studies (QUADAS-2) guidelines was used [16]. It is constituted of four domains: (1) patient selection, (2) index test, (3) reference standard and (4) flow and timing. The risk of bias and concerns about applicability were rated as low risk, high risk and unclear risk. QUADAS-2 was performed by Review Manager 5.3.

## Results

### Study selection

**Fig 1:**
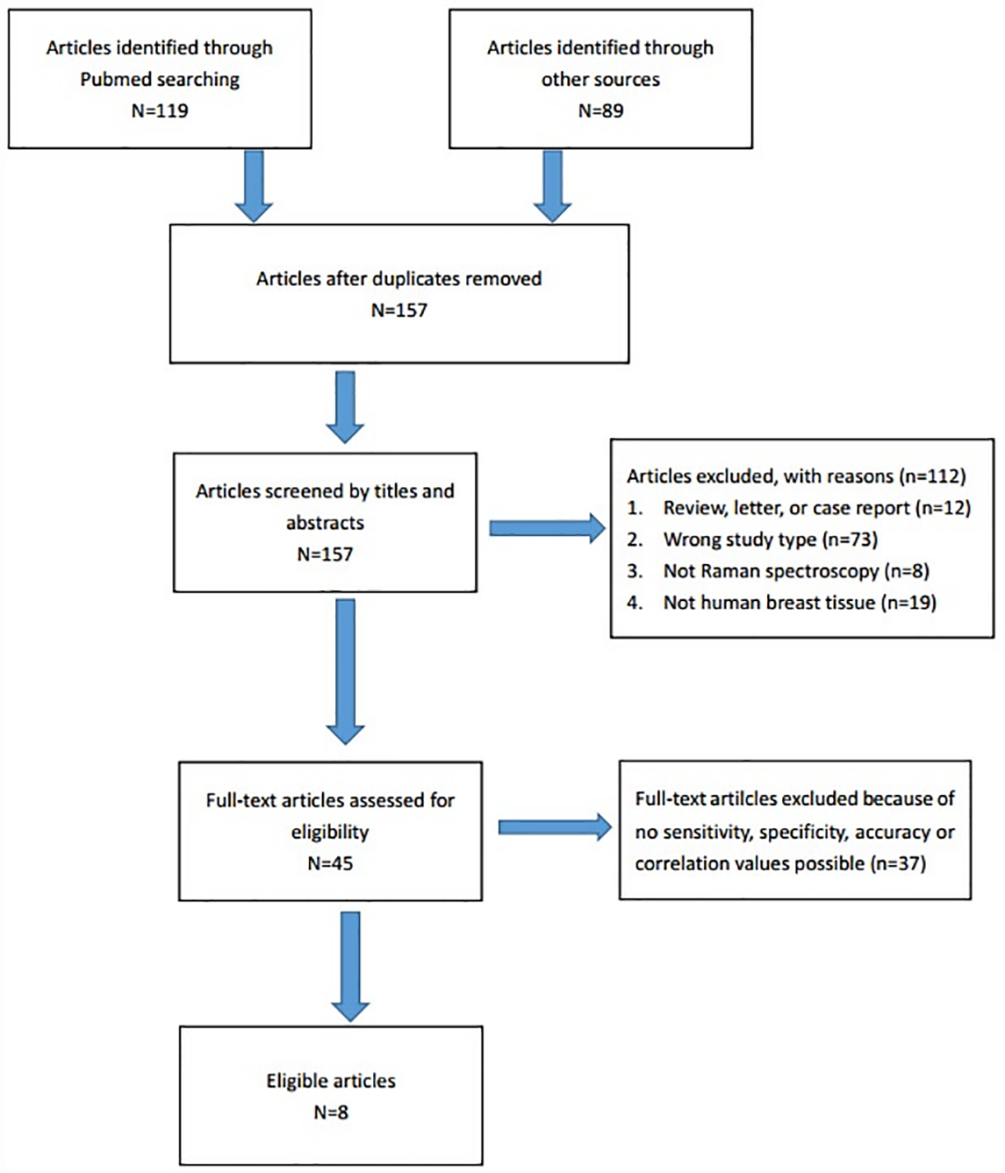
The full screening and selection process.

After an initial literature search, we identified a total of 157 articles. All of them were written in English. Then, 112 articles were excluded by manual screening of the titles and abstracts. Full texts and data integrity of the remaining 45 articles were reviewed for more detailed evaluation and 36 articles were further excluded. Ultimately, nine studies [17–25] with ten groups of data were eligible in this meta-analysis according to the inclusion criteria. Studies performed by the same author(s) of which the population sources were distinct from each other [19, 20, 25] were included. Two groups of data were extracted from one article [17] because they used different diagnostic algorithms and the data differed from each other with the same study samples. No further studies were identified by checking the “related articles” in the reference lists of the relevant publications. The full screening and selection process is shown in Fig 1.

### Study characteristics

Among the nine eligible studies, 8 studies with a total of 1756 breast lesions investigated the diagnostic performance of RAS in vitro, and only 1 study was performed in vivo [25] which presented a malignant sample out of a total of 30 samples in the dataset. All eligible studies were published in English. Most of the studies were conducted in the United States (n = 6) among which three of them were conducted by the same research team. Others were conducted in China (n = 1), Poland (n = 1), and UK (n = 1). All the above studies applied pathological diagnosis as the golden standard. Various statistical models including fit coefficients (FC), support vector machine (SVM), principal component analysis (PCA) and their derived diagnostic algorithms were explored and performed to discriminate breast lesions (Table 1).

**Table 1:** Characteristics of the 8 in vitro studies included in the meta-analysis.

Author	Year	Nation	N1	N2	ST	TP	FP	FN	TN	laser(nm)	Study design#
Surmacki J	2015	Poland	82	100	Fre	74	3	8	15	532	#
Haka AS	2005	USA	58	126	Fro	29	4	2	91	830	+
Majumder SK	2008	USA	74	293	Fro	102	1	2	188	785	+
Haka AS	2009	USA	21	129	Fre	5	9	1	114	830	+
Keller MD	2011	USA	NR	35	Fro	19	0	1	15	785	#
Barman I	2013	USA	33	146	Fre	10	0	6	130	830	+
Hu C*	2013a	China	168	300	Fre	85	21	15	179	785	+
Hu C*	2013b	China	168	300	Fre	95	7	5	193	785	+
Kong K	2014	UK	60	627	Fro	311	12	13	291	785	#

N1, number of patients; N2, number of spectra; ST, sample type; TP, true positives; FP, false positives; FN, false negatives; TN, true negatives; Fre, fresh tissue; Fro, frozen tissue.

^#^ The study did not include samples of fibroadenoma.

^+^ The study included samples of fibroadenoma.

*Two groups of data were extracted from one article because of the usage of different diagnostic algorithms.

### Overall analysis

**Fig 2:**
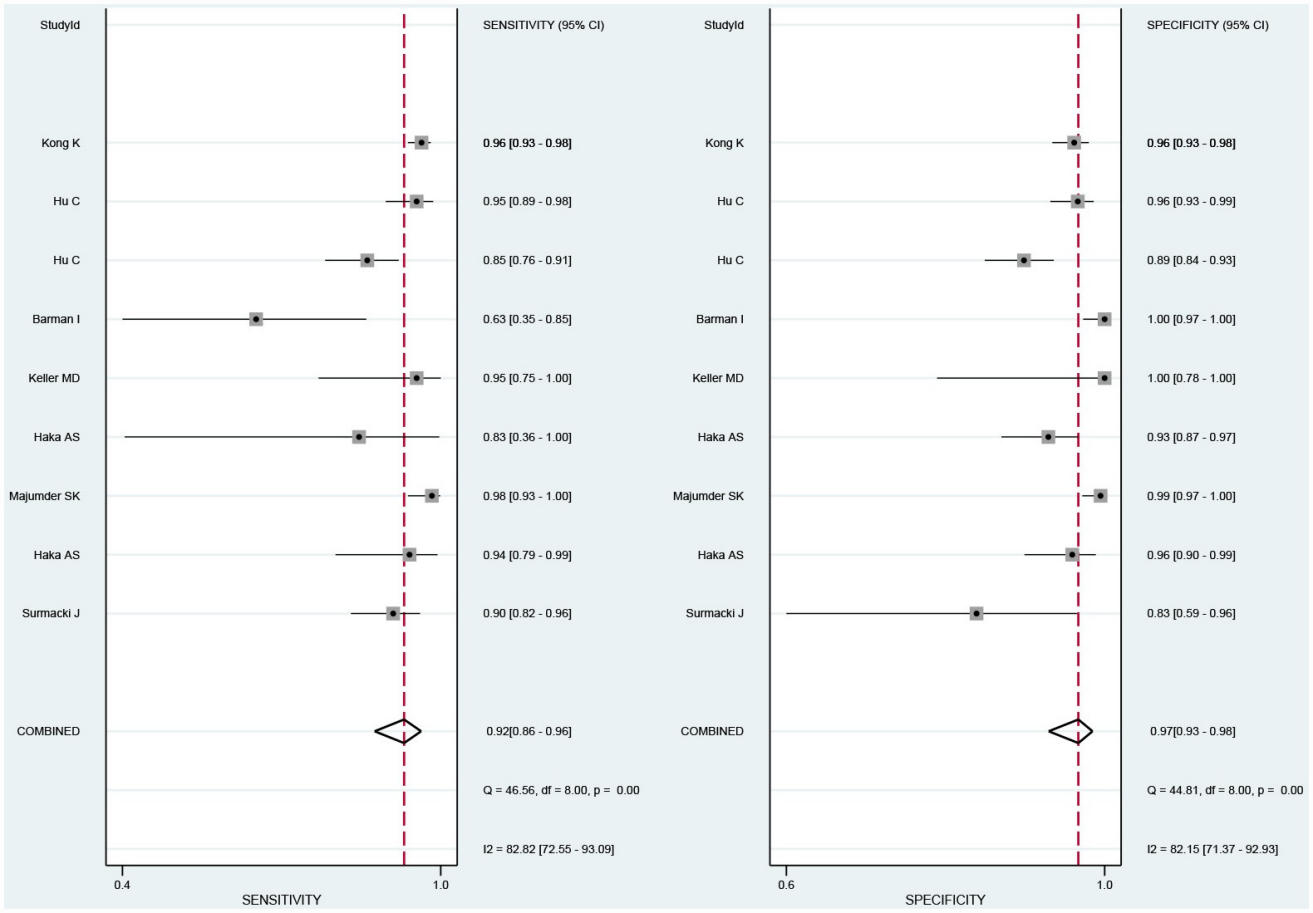
The pooled sensitivity and specificity of Raman spectroscopy system (RAS).

**Fig 3:**
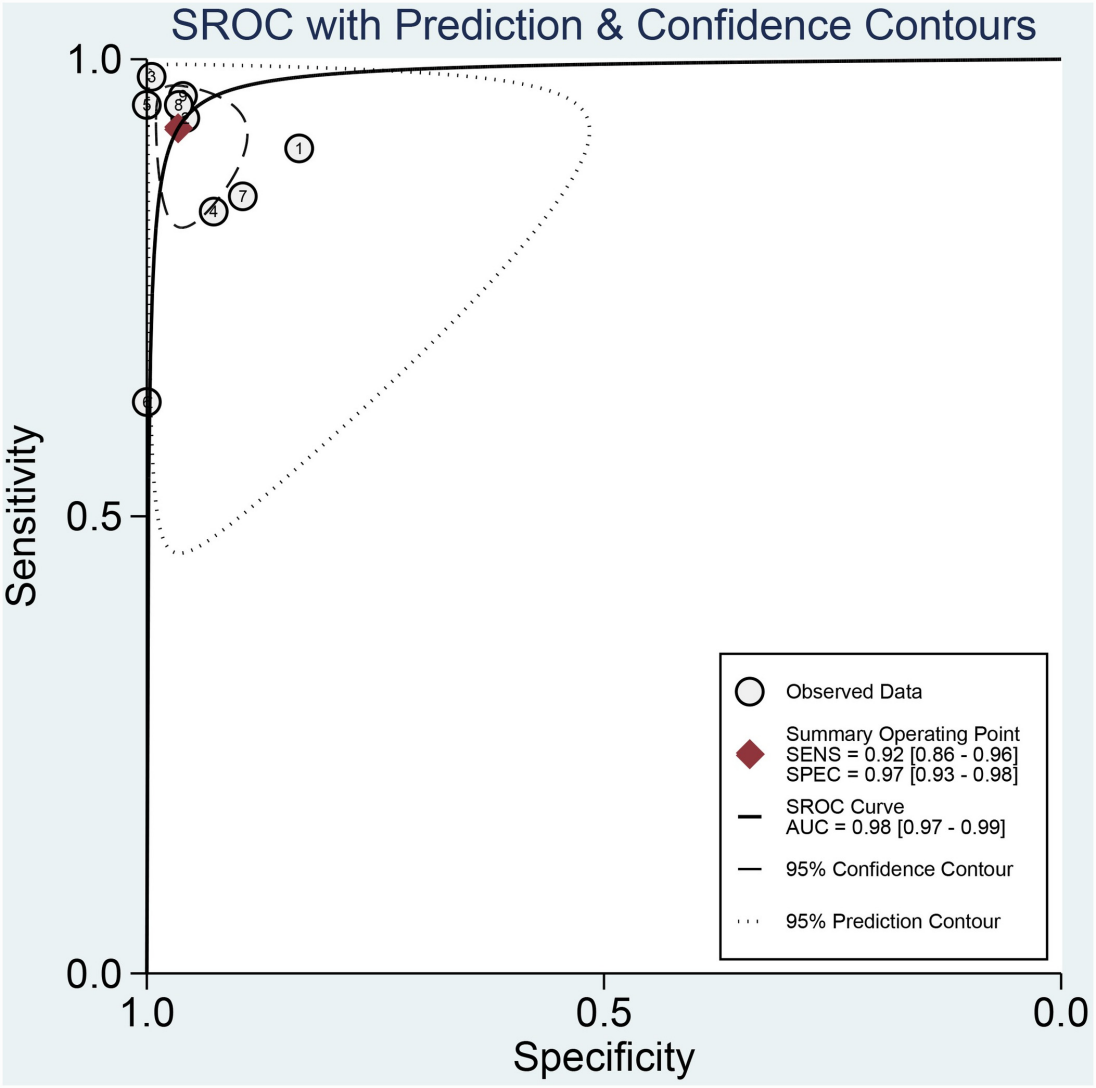
The area under the curve (AUC) of SROC (summary receiver operating characteristic) curves.

We pooled and analyzed the data of eight in vitro studies separately from the in vivo one. Consequently, all pooled parameters presented in this meta-analysis were derived from in vitro studies. The sensitivity, specificity, PLR, NLR, and diagnostic odds ratio (DOR) were reckoned to measure the overall diagnostic accuracy. The pooled sensitivity and specificity of RAS were 0.92 (95% CI 0.86–0.96) and 0.97 (97% CI 0.93–0.98), respectively (Fig 2). The pooled PLR and NLR were 18.91 (95% CI 10.09–35.45) and 0.09 (95% CI 0.05–0.18), respectively. The DOR of RAS was 266.70 (95% CI 89.38–795.79), demonstrating high accuracy. The area under the curves (AUC) of SROC (summary receiver operating characteristic) curves was 0.98 (95% CI 0.97–0.99) (Fig 3). The heterogeneity was significant among all pooled in vitro studies (I^2^>50%, P < 0.05)

### Subgroup analysis

For studies using samples of non-Asian patients, a random effect model was applied to calculate DOR because heterogeneity existed between the studies. Although the parameters for Asian patients could not be calculated as there were only two groups of data, we found diagnostic performance of RAS was better for non-Asian patients than for all patients, with DOR being 334.00 (I^2^ = 67.9%, P = 0.0047). Considering the size of corresponding spectra may be a source of heterogeneity, we stratified all the studies into two groups, one for studies with less than 200 spectra and the other for studies with no less than 200 spectra. The results indicated that the size of corresponding spectra contributed to heterogeneity as the DOR of the smaller group was 129.70 (I^2^ = 11.8%, P = 0.3382). We also noticed larger spectra size was significantly correlated with elevated DOR, with DOR being 482.70 (I^2^ = 91.3%, P = 0) in the larger group with no less than 200 spectra. Then subgroup analysis was performed in frozen tissues and fresh tissues. In our study, fresh tissue referred to breast tissue which had not been snap-frozen in liquid nitrogen for storage and spectra were obtained within 30 minutes of excision. Significantly higher sensitivity, specificity and DOR were observed in studies using frozen tissues, which were 0.96 (I^2^ = 0.0%, P = 0.6100), 0.97 (I^2^ = 63.1%, P = 0.0433) and 703.91 (I^2^ = 46.0%, P = 0.1351), respectively, and fresh tissue was associated with lower DOR (112.09, I^2^ = 70.6%, P = 0.0087). In addition, heterogeneity disappeared in the frozen tissues group.

Diagnostic algorithms of RAS are significant methods applied to discriminate the target tissue [26]. Although they varied among the included studies, we managed to create subgroups by assessing whether the diagnostic algorithm was based on PCA or not. A fixed effect model was used to pool DOR of studies without utilizing PCA because the heterogeneity was statistically insignificant (I^2^<50%, P > 0.05). We found that DOR of non-PCA group was 566.44 (46.7%, P = 0.0947), which demonstrated higher diagnostic accuracy compared with PCA group (DOR 112.92, 91.3%, P = 0). We failed to analyze other algorithms for lack of adequate data. When we grouped by diode laser wavelength, studies using laser 830nm and 785nm were assessed separately. Laser 830nm resulted in a relatively low level of sensitivity (0.83, I^2^ = 70.9%, P = 0.0320) and DOR (223.05, 0.0%, P = 0.4554). Moreover, we grouped all studies according to the type of study design which was defined by whether samples of fibroadenoma were included. The results of subgroup analysis of all studies in our meta-analysis are shown in Table 2.

**Table 2:** The results of subgroup analysis of all studies in our meta-analysis.

Subgroups	No.of studies	Groups of data	No.of spectra	SEN(I^2^,P-value,model)	SPE(I^2^,P-value,model)	PLR(I^2^,P-value,model)	NLR(I^2^,P-value,model)	DOR(I^2^,P-value,model)
All studies	8	9	1756	0.93(75.7%,P = 0.0001,REM)	0.96(81.5%,P = 0,REM)	18.91(76.8%,P = 0,REM)	0.09(83.9%,P = 0,REM)	266.70(80.2%,P = 0,REM)
Race								
Asia	1	2	300					
Non-Asia	7	7	1456	0.94(73.9%,P = 0.0008,REM)	0.97(78.0%,P = 0.0001,REM)	21.66(68.6%,P = 0.0040,REM)	0.09(86.2%,P = 0,REM)	334.00(67.9%,P = 0.0047,REM)
No.of spectra								
<200	5	5	536	0.88(58.1%,P = 0.0486,REM)	0.96(78.9%,P = 0.0008,REM)	13.72(47.5%,P = 0.1064,FEM)	0.15(64.8%,P = 0.0229,REM)	129.70(11.8%,P = 0.3382,FEM)
> = 200	3	4	1220	0.94(82.4%,P = 0.0007,REM)	0.95(87.5%,P = 0,REM)	24.26(88.5%,P = 0,REM)	0.06(86.7%,P = 0.0001,REM)	482.70(91.3%,P = 0,REM)
Sample type								
Fresh tissue	4	5	675	0.88(71.2%,P = 0.0077,REM)	0.94(85.4%,P = 0,REM)	12.64(71.3%,P = 0.0075,REM)	0.15(76.6%,P = 0.0018,REM)	112.09(70.6%,P = 0.0087,REM)
Frozen tissue	4	4	1081	0.96(0.0%,P = 0.6100,FEM)	0.97(63.1%,P = 0.0433,REM)	31.49(33.6%,P = 0.2106,FEM)	0.04(0.0%,P = 0.5045,FEM)	703.91(46.0%,P = 0.1351,FEM)
Diagnostic algorithm								
PCA	3	3	1027	0.93(85.3%,P = 0.0011,REM)	0.93(80.5%,P = 0.0059,REM)	10.71(85.5%,P = 0.0010,REM)	0.09(87.9%,P = 0.0003,REM)	112.92(91.3%,P = 0,REM)
Non-PCA	6	6	1029	0.94(73.7%,P = 0.0019,REM)	0.97(76.2%,P = 0.0008,REM)	31.32(70.5%,P = 0.0046,REM)	0.09(85.2%,P = 0,REM)	566.44(46.7%,P = 0.0947,FEM)
Laser wavelength								
laser = 830nm	3	3	401	0.83(70.9%,P = 0.0320,REM)	0.96(85.1%,P = 0.0012,REM)	21.56(67.5%,P = 0.0463,REM)	0.18(72.8%,P = 0.0252,REM)	223.05(0.0%,P = 0.4554,FEM)
Others	5	6	1355	0.94(73.7%,P = 0.0019,REM)	0.95(82.8%,P = 0,REM)	18.60(83.0%,P = 0,REM)	0.07(80.0%,P = 0.0001,REM)	307.65(87.1%,P = 0,REM)
laser = 785nm	4	5	1255	0.94(76.5%,P = 0.0019,REM)	0.95(84.3%,P = 0,REM)	24.50(84.9%,P = 0,REM)	0.06(82.2%,P = 0.0002,REM)	466.87(88.5%,P = 0,REM)
Others	4	4	501	0.87(62.4%,P = 0.0388,REM)	0.96(83.0%,P = 0.0005,REM)	13.89(58.2%,P = 0.0666,REM)	0.17(69.1%,P = 0.0212,REM)	115.69(24.2%,P = 0.2661,FEM)
Study design								
+	5	6	994	0.91(80.3%,P = 0.0001,REM)	0.96(86.8%,P = 0,REM)	23.24(82.4%,P = 0,REM)	0.10(84.8%,P = 0,REM)	312.27(81.9%,P = 0,REM)
#	3	3	762	0.95(47.1%,P = 0.1510,FEM)	0.96(62.7%,P = 0.0685,REM)	14.10(69.3%,P = 0.0384,REM)	0.07(70.5%,P = 0.0338,REM)	211.72(78.0%,P = 0.0106,REM)

^#^ The study did not include samples of fibroadenoma.

^+^ The study included samples of fibroadenoma.

SEN, sensitivity; SPE, specificity; PLR positive likelihood ratio; NLR, negative likelihood ratios; DOR, diagnostic odds ratio; REM, random effects model; FEM, fixed effects model; PCA, principal component analysis.

### Assessment of study quality and publication bias

**Fig 4:**
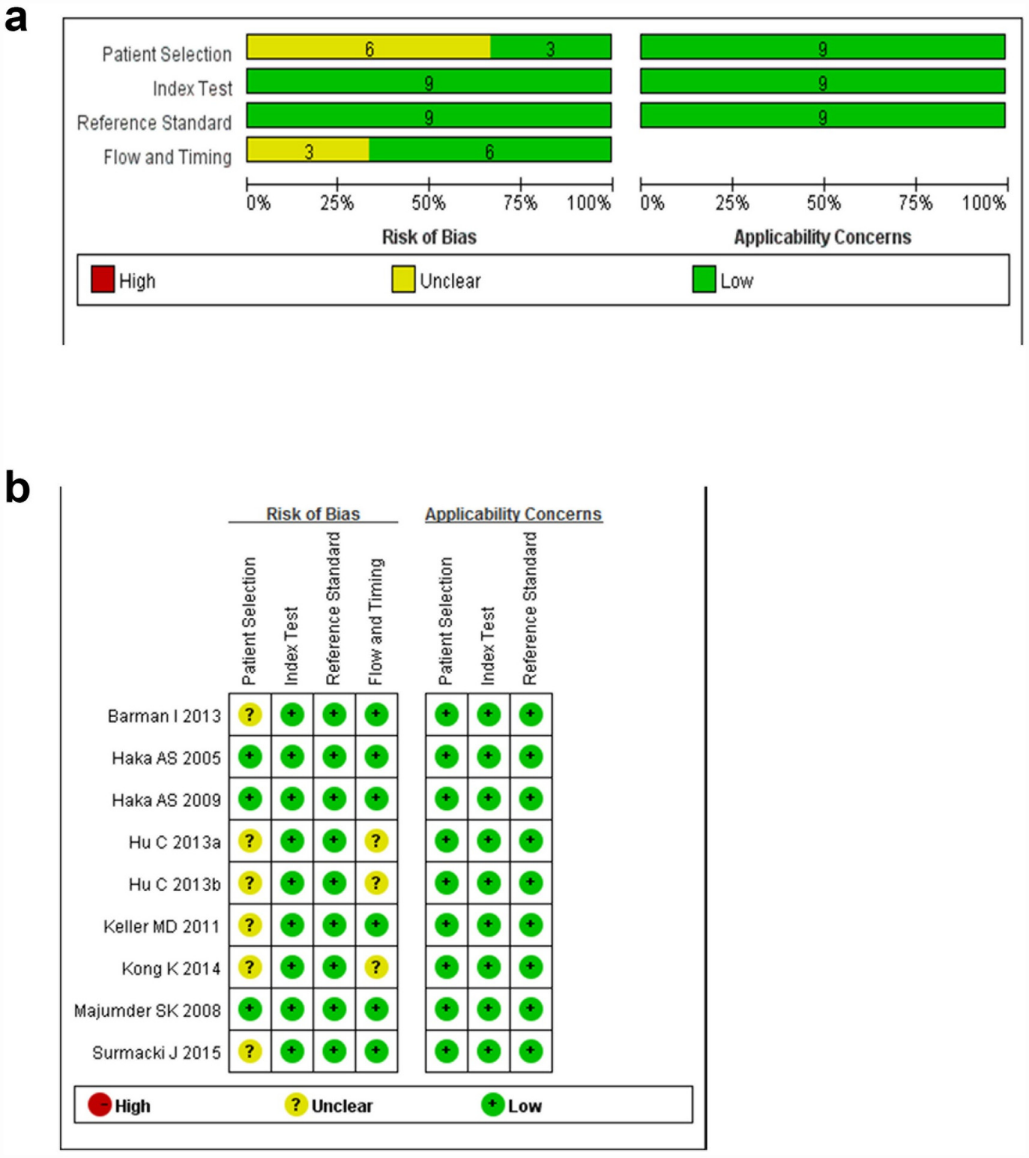
The graphical display of the evaluation of the risk of bias and concerns regarding applicability of the selected studies. (a) Risk of bias and applicability concerns evaluation of included studies in pool. (b) Risk of bias and applicability concerns evaluation of included studies individually.

**Fig 5:**
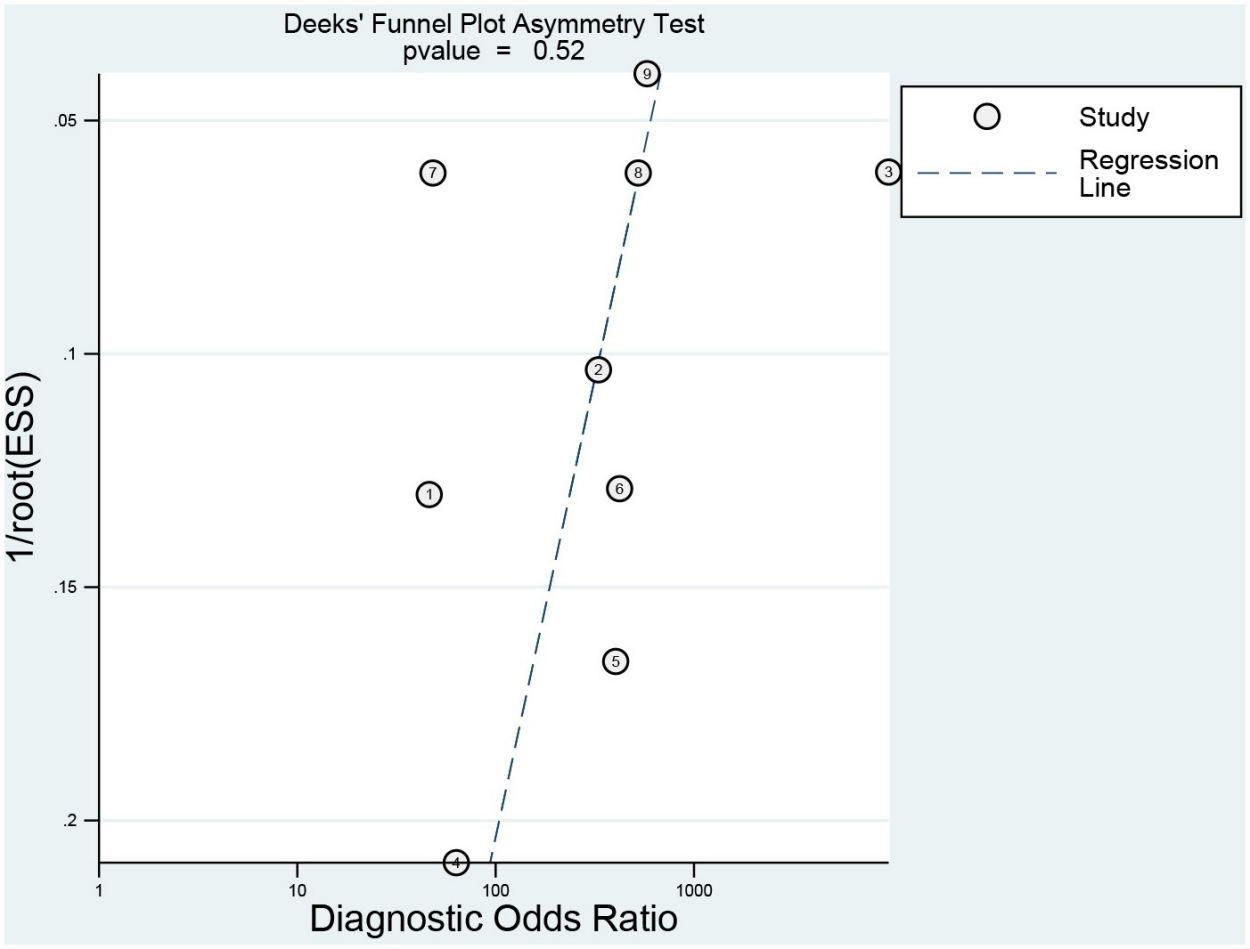
The Deeks' funnel plot asymmetry test.

Two reviewers evaluated the methodological quality of each study according to the QUADAS-2 guidelines [16] independently. All QUADAS-2 items were used to evaluate the eligible articles. The graphical display of the evaluation of the risk of bias and concerns regarding applicability of the selected studies were shown in Fig 4. The Deeks' funnel plot asymmetry test was performed to evaluate publication bias in the included studies and the result indicated that no significant publication bias was found (P = 0.52) (Fig 5).

## Discussion

The technique of RAS has been widely explored in recent years. Here, with the aim of summarizing the evidences on the accuracy of RAS in the detection of breast cancer lesions, we presented the first meta-analysis which systematically investigated the diagnostic performance of RAS according to the standard protocol for a systematic review. Nine studies with ten groups of data were included in our work. It is worth pointing out that in spite of inadequate positive sample size, Haka’s study [25] was the only one conducted in vivo and it clearly suggested the feasibility of Raman spectroscopy in clinical use, such as real-time margin assessment during BCS. Thus we reviewed it separately to underline the importance of conducting more studies to explore the application of RAS in breast tissues in vivo.

The meta-analysis provided evidence for good diagnostic accuracy of RAS in differentiating breast lesions, with the overall AUC being 0.98 (95% CI 0.97–0.99). It was in agreement with the recent meta-analysis [10]. We detected obvious heterogeneity within all pooled studies, so we performed subgroup analyses in order to investigate the potential factors that influence the heterogeneity and diagnostic accuracy. The type of sample (in or ex vivo) was suggested to be put into consideration by a previous meta-analysis [10]. Nevertheless, we were not able to analyze it because only one study with 30 samples performed in vivo was included. Data of different types of samples (fresh or frozen) was tested separately as frozen-thawed tissues were not perfect surrogates for freshly excised tissue and their optical properties differed from each other [27, 28]. And considering that the sensitivity of studies on diagnostic accuracy can be underestimated if the sample size is relatively small, we subdivided the studies to assess if the size of corresponding spectra was the causing factor of heterogeneity. Different patients’ demographic and clinical characteristics, different instruments, measurements, and experimental design could also contribute to heterogeneity. Based on those considerations, we grouped the studies by the race of patients (Asian or non-Asian), diagnostic algorithms (PCA or others), laser wavelength, and study design due to the diversity of these characteristics and the limited number of studies.

In the subgroup analyses, we found that the size of corresponding spectra (≥200 or <200), the type of sample (fresh or frozen), diagnostic algorithms (PCA or others), and laser wavelength were the sources of heterogeneity. Although whether experiment was conducted in non-Asian patients or Asian patients was not the source of heterogeneity as it was not eliminated in subgroup analyses, RAS performed better in non-Asian patients than in Asian patients. We noticed the two studied performed in Asian patients were conducted by the same research group and the only difference between those two studies is the diagnostic algorithm they used. So any factor except the diagnostic algorithm can contribute to the difference. To address the underlying cause, we are expecting data from more studies with larger sample size to confirm the results and provide enough information to do more detailed subgroup analysis. According to our results, RAS performed better in frozen tissue than in freshly excised tissue. However, it doesn’t necessarily hint that RAS is not suitable to be done in vivo other than in vitro. As it’s more valuable to apply this technique to be used for real-time intra-operatory tumor margin detection, further researches need to be conducted to figure out why frozen tissues performed better than fresh tissues and push forward the development of the technique. Furthermore, RAS presented better diagnostic performance when the number of spectra within each study was no less than 200. Pooled sensitivity was obviously decreased when studies of less than 200 spectra were included in the subgroup studies. It is possible there’s a learning curve limitations in applying this technique as we found most of included studies didn’t report detailed information including the size and depth of breast lesions they analyzed and those factors could affect the accuracy of detection and to some extend be decided by operators’ experience. Meanwhile, underestimated sensitivity of diagnostic studies with small sample size may also be a reasonable interpretation for the result. The subgroup analysis also indicated that PCA is not positively related to better diagnostic performance and laser 830nm might lead to a relatively low level of sensitivity. The potential reasons remain unclear. Thus, further studies are still needed to both confirm and explain the results. Moreover, when we took study design into account as described before, we noticed the group with fibroadenoma performed slightly better than the group without fibroadenoma. Data from the included studies was limited and various factors possessed the possibility to affect the result, but we inferred when similar tissues needed to be distinguished, RAS may be more accurate.

We acknowledge that our study has several potential limitations. First, both the quantity of included studies and the amount of samples are relatively small because of the limited published researches in this area. Second, the selection of the samples and patients are usually not made by blind test method which might add to the bias of the data. Third, six out of our nine included studies showed unclear risk of bias in terms of patient selection. Fourth, a variety of factors could have contributed to the heterogeneity and we were not able to analyze all of them. Fifth, we didn’t manage to perform meta-analysis in various pathologic subtype of breast cancer owing to the lack of available data. Although all the included studies reported the pathologic type of their tissues, they did not provide detailed testing result for each tissue. Instead, researchers managed all breast carcinoma as a single distinguished population and looked for the difference between malignant tissue and benign or normal tissue. Sixth, we could not analyze the size and depth of breast lesions which may have effects on the accuracy of detection [29].

In summary, RAS has been demonstrated to be a reliable testing approach for in vitro breast tumor margin detection with high accuracy. Meanwhile, it’s very promising to be investigated and applied for in vivo detection. Although various factors can impact the diagnostic performance of RAS and some of them haven’t been well understood, it is worthy of being further explored and developed to be a clinical diagnostic tool for real-time intra-operatory tumor margin detection.

## Supporting Information

S1 Appendix Literature searching strategy including searching details. (PDF)

S1 Checklist PRISMA-IPD Checklist of items to include when reporting a systematic review and meta-analysis of individual participant data (IPD) [30]. (PDF)
