# Supplementary material for: Rapid Discrimination of Malignant Breast Lesions from Normal Tissues Utilizing Raman Spectroscopy System: A Systematic Review and Meta-Analysis of In Vitro Studies
Source: PLoS One. 2016 Jul 26;11(7):e0159860. doi: 10.1371/journal.pone.0159860 (PMC4961451; doi:10.1371/journal.pone.0159860)
Supplement: S1 Appendix — (PDF) [file pone.0159860.s001.pdf]

Appendix

Pubmed search 2000 to June 2015

1. Search term combination of "breast", "cancer", "Raman", "spectra" (("breast"[MeSH Terms] OR "breast"[All Fields]) AND ("neoplasms"[MeSH Terms] OR "neoplasms"[All Fields] OR "cancer"[All Fields]) AND Raman[All Fields] AND spectra[All Fields]) AND ("2000/01/01"[PDAT] : "2015/06/31"[PDAT])- **88 articles**
2. Search term combination of "breast", "sensitivity", "Raman", "spectra" (("breast"[MeSH Terms] OR "breast"[All Fields]) AND ("sensitivity and specificity"[MeSH Terms] OR ("sensitivity"[All Fields] AND "specificity"[All Fields]) OR "sensitivity and specificity"[All Fields] OR "sensitivity"[All Fields]) AND Raman[All Fields] AND spectra[All Fields]) AND ("2000/01/01"[PDAT] : "2015/06/31"[PDAT])- **31 articles**

Embase search 2000 to June 2015

1. Search keywords combination of "Raman spectrometry" and "breast cancer" Field: Title/Abstract, Limits: Humans- **89 articles**
